# Supplementary material for: Responses of the putative trachoma vector, Musca sorbens, to volatile semiochemicals from human faeces
Source: PLoS Negl Trop Dis. 2020 Mar 3;14(3):e0007719. doi: 10.1371/journal.pntd.0007719 (PMC7069642; doi:10.1371/journal.pntd.0007719)
Supplement: S1 Table — (DOCX) [file pntd.0007719.s001.docx]

**Table S1. Median female *Musca sorbens* caught per trap per 24-hours (IQR) in The Gambia (Boiram and Farafenni) and Ethiopia (Oromia).**

|  |  | Study Location |  |
| --- | --- | --- | --- |
| Bait | Boiram | Farafenni | Oromia |
| Calf | 1 (0-3) | 2 (1-3) |  |
| Cow | 1 (0-1) | 2 (0-2.5) | 0 (0-1) |
| Donkey | 0 (0-1) | 1 (0-2) | 0 (0-1) |
| Dog |  | 21 (11-25.5) |  |
| Empty pot | 0 (0-0) |  |  |
| Horse | 1 (0-2) | 4 (1-5.5) |  |
| Sheep | 1 (0-3) | 2 (0-5) |  |
| Human | 10 (6-25) | 63.5 (48.5-83.5) |  |
| Human adult |  |  | 6 (0-8) |
| Human child |  |  | 2 (0-8) |
| Soil (Baseline) | 0 (0-0) | 1 (0-2) | 0 (0-1) |
