# Supplementary material for: Responses of the putative trachoma vector, Musca sorbens, to volatile semiochemicals from human faeces
Source: PLoS Negl Trop Dis. 2020 Mar 3;14(3):e0007719. doi: 10.1371/journal.pntd.0007719 (PMC7069642; doi:10.1371/journal.pntd.0007719)
Supplement: S2 Table — (DOCX) [file pntd.0007719.s002.docx]

**Table S2. Median male *Musca sorbens* caught per trap per 24-hours (IQR) in The Gambia (Boiram and Farafenni) and Ethiopia (Oromia).**

|  |  | Study Location |  |
| --- | --- | --- | --- |
| Bait | Boiram | Farafenni | Oromia |
| Calf | 0 (0-0) | 0 (0-0) |  |
| Cow | 0 (0-1) | 0 (0-0) | 0 (0-0) |
| Donkey | 0 (0-0) | 0 (0-0.5) | 0 (0-0) |
| Dog |  | 0 (0-1) |  |
| Empty pot | 0 (0-0) |  |  |
| Horse | 0 (0-1) | 0 (0-0) |  |
| Sheep | 0 (0-1) | 0 (0-1) |  |
| Human | 1 (0-3) | 2.5 (0-3) |  |
| Human adult |  |  | 0 (0-1) |
| Human child |  |  | 0 (0-0) |
| Soil (Baseline) | 0 (0-1) | 0 (0-0) | 0 (0-1) |
