# Supplementary material for: Responses of the putative trachoma vector, Musca sorbens, to volatile semiochemicals from human faeces
Source: PLoS Negl Trop Dis. 2020 Mar 3;14(3):e0007719. doi: 10.1371/journal.pntd.0007719 (PMC7069642; doi:10.1371/journal.pntd.0007719)
Supplement: S3 Table — (DOCX) [file pntd.0007719.s003.docx]

**Table S3. Odds that *Musca sorbens* caught by different bait types would be female, relative to soil control, in The Gambia (Boiram and Farafenni) and Ethiopia (Oromia).**

| Study | Bait | Odds ratio (95 % CI) | *P*-value* |
| --- | --- | --- | --- |
| Boiram | Calf | 5.75 (1.12-29.41) | 0.036* |
|  | Cow | 2.20 (0.43-11.22) | 0.343 |
|  | Empty pot | 1.17 (0.22-6.08) | 0.855 |
|  | Horse | 1.78 (0.40-7.84) | 0.447 |
|  | Human | 7.93 (2.16-29.10) | 0.002* |
|  | Sheep | 3.25 (0.75-14.15) | 0.116 |
| Farafenni | Calf | 6.00 (1.10-32.76) | 0.039* |
|  | Cow | 3.44 (0.77-15.48) | 0.107 |
|  | Dog | 17.71 (5.40-58.15) | <0.001** |
|  | Donkey | 1.47 (0.38-5.60) | 0.575 |
|  | Horse | 22.00 (2.49-194.66) | 0.005* |
|  | Human | 11.52 (4.29-30.96) | <0.001** |
|  | Sheep | 2.80 (0.76-10.37) | 0.123 |
| Oromia | Human adult | 14.00 (3.63-53.99) | <0.001** |
|  | Human child | 32.67 (5.59-191.06) | <0.001** |
|  | Cow | 10.67 (1.03-109.94) | 0.047* |
|  | Donkey | 2.67 (0.47-15.25) | 0.270 |

* Significant at 5 % level

** Significant at 1 % level
