# Supplementary figures and images for: Responses of the putative trachoma vector, Musca sorbens, to volatile semiochemicals from human faeces
Source: PLoS Negl Trop Dis. 2020 Mar 3;14(3):e0007719. doi: 10.1371/journal.pntd.0007719 (PMC7069642; doi:10.1371/journal.pntd.0007719)

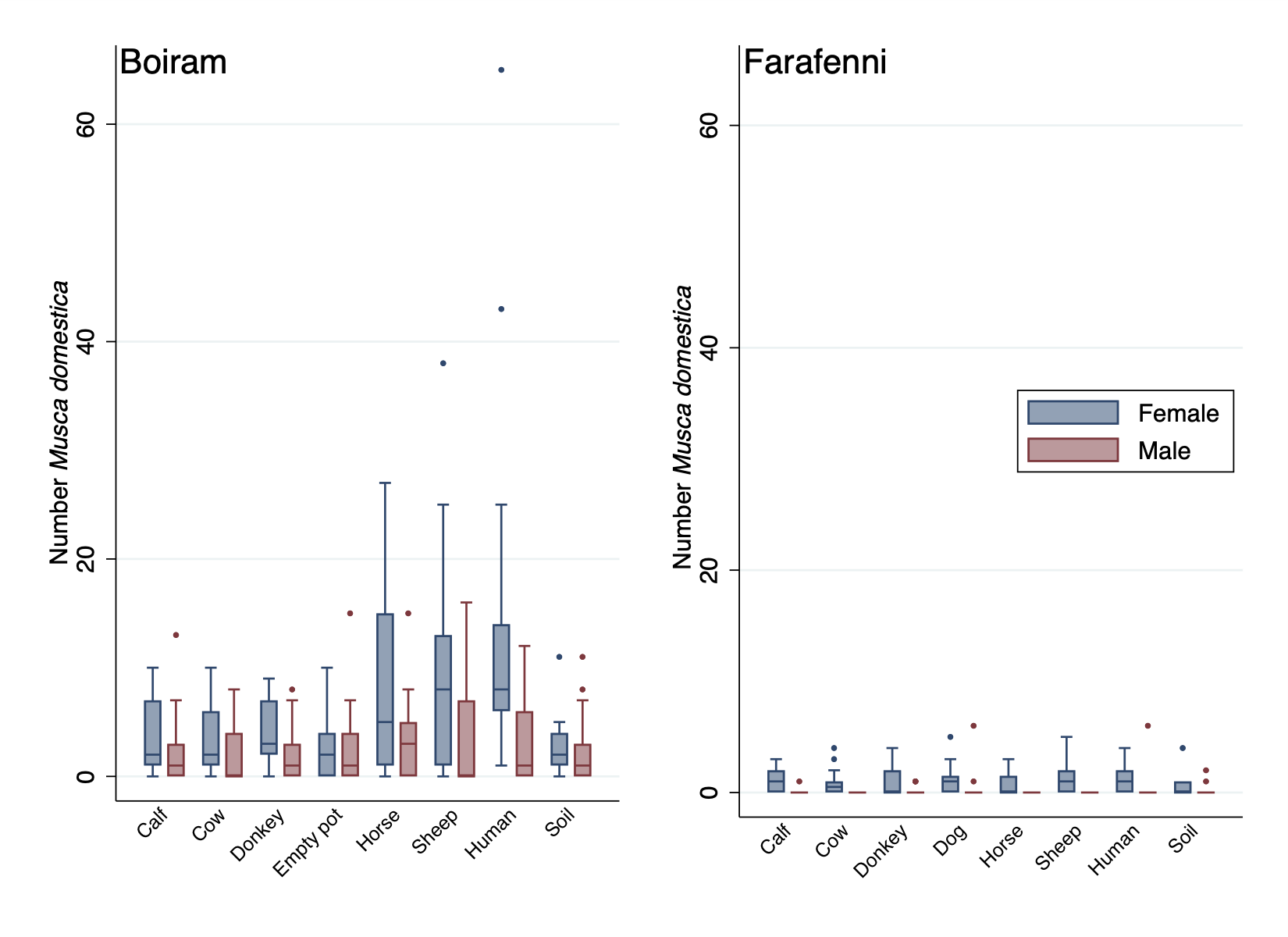

Supplement: S1 Fig — Trap days per bait are Boiram, n = 16; Farafenni, n = 16 (sheep n = 15). (TIF) [file pntd.0007719.s005.tif]
