# Supplementary material for: Responses of the putative trachoma vector, Musca sorbens, to volatile semiochemicals from human faeces
Source: PLoS Negl Trop Dis. 2020 Mar 3;14(3):e0007719. doi: 10.1371/journal.pntd.0007719 (PMC7069642; doi:10.1371/journal.pntd.0007719)
Supplement: Attachment — Submitted filename: robinson_rebuttal_20191213.docx [file pntd.0007719.s007.docx]

London School of Hygiene & Tropical Medicine,

Keppel Street,

London

WC1E 7HT

13^th^ December 2019

Dear Editor and Referees,

Thank you very much for taking the time to review our manuscript “Responses of the putative trachoma vector, *Musca sorbens*, to volatile semiochemicals from human faeces”, for submission to PLOS Neglected Tropical Diseases. We appreciate the helpful comments and suggestions that were proposed by the referees. In response to these, we have amended the manuscript appropriately and feel that it has benefitted from this process. Please see below for point-by-point responses to the referee comments; note that the line numbers refer to the track-changes document.

Yours sincerely,

Ailie Robinson and co-authors

[Ailie.robinson@lshtm.ac.uk](mailto:Ailie.robinson@lshtm.ac.uk)

London School of Hygiene and Tropical Medicine

00447885679592

# Response to reviewers

## Reviewer 1

**Methods**

Reviewer #1:

1. Objectives could have been more strongly stated.
   a. *Agreed, and in response to this comment we have modified the text (L146-149)*
2. A trapping system was developed to capture adult flies and flies were reared to produce adults for the GC-EAG studies. Description of traps is confusing. One complete trap design should be described, then the modifications made to arrive at the next design and so on.
   a. *We have modified the trap description to start with the basic design (for clarity) before detailing specifics and differences in design between the two studies. Further, we have added dimensions to the schematic image of the trap, including a photo of a deployed trap. (L190-199)*
3. Line 165 - sounds like the yellow sticky trap is really a yellow disc coated with adhesive, but do not know.
   a. *‘Sticky trap’ (/sticky paper) is the name conventionally used for sheets of card coated with adhesive, commercially available from many sources. The two referred to in these studies are available here* [*https://www.agralan.co.uk/biological-pest-control/traps-sticky-traps/yellow-sticky-traps-pk-7.html*](https://www.agralan.co.uk/biological-pest-control/traps-sticky-traps/yellow-sticky-traps-pk-7.html) *and here* [*http://www.agrisenseuk.co.uk/medium-yellow-sticky-trap/?doing_wp_cron=1515848574.5228850841522216796875*](http://www.agrisenseuk.co.uk/medium-yellow-sticky-trap/?doing_wp_cron=1515848574.5228850841522216796875). *We have amended the text to state that circles were cut from this ‘sticky trap’ paper (L193)*
4. Lines 166-167 - Nylon and polyester mesh used to prevent entry of flies into the traps, correct? Is there a mesh size to be used?
   a. *The mesh (gauge) size used in The Gambia is known (L195), the gauge size of the mesh used in Ethiopia is unknown and so we have stated this (L196).*
5. Line 170 - which company manufacturers Rat Stop?
   a. *We managed to establish who the manufacturers were (Taizhou Aiyon Adhesive Co., China) and have amended as such (L202)*
6. Lines 172-173 - Wire frames. Made from individual wires, pieces of wire fence or what? Coated in black electrical tape? Size of frames given in cubic cm. This can be many shapes. Need length, width and height.
   a. *We have expanded our description of the wire frames to include dimensions and the overall shape (L205-206).*
7. Discussion of feces collections do not strongly state how fresh the feces must be to qualify for the studies.
   a. *Although there were no technical measures/criteria for faeces freshness (or moisture content), only fresh samples were used to ensure maximum volatile release. Only faeces that were less than one day old were used (L371-372)*
8. Line 196 - soil from uncontaminated areas. Contaminated with what? Collected near trap sites. How near or far?
   a. *By this we meant uncontaminated with faeces, however we agree the sentence was unclear. We have amended this, and indicated the distance of ‘control’ soil bait collection from any possible faecal contamination (L381-382)*
9. Moisture content of feces would be interesting to know.
   a. *We agree with this reviewer’s comment. Although this was not done in the present study, further work to establish for how long faeces remain attractive may be an interesting additional piece of work, and this could indeed be estimated using moisture content. We have added this in the discussion (L801-807).*
10. Line 201 - traps were separated by 50 cm. This seems a bit close.
    a. *Although 0.5 m may appear quite close, in the environment where these traps were deployed there are often multiple faeces sources, both of humans and animal origin, in the immediate vicinity. This is generally the case where* M. sorbens *are prevalent, and represents a realistic setting, therefore our experimental design allowed us to test which competing faecal odour is the most attractive. The results presented illustrate that the odour of human faeces is preferred, and the control bait (soil) was not attractive. If nearby traps had interfered with one another, it is unlikely that we would have seen this result. We have improved our discussion by adding this (L772-774).*
11. Line 227 - was the portable air entrainment kit made by the authors, or a commercial model?
    a. *The air entrainment kits used were made by a colleague at one of the institutions, Rothamsted Research. This information has been added (L432).*
12. Fly rearing paragraph could be more clearly stated. Line 252 - need dimensions of BugDorms, not cubic cm. How many BugDorms and how many flies in each?
    a. *We have added the correct dimensions for the BugDorm cages (L451)*
13. Line 253 - what is blue roll?
    a. *We mean blue paper towelling (on a roll), clarified (L452)*
14. Lines 253-254 - on a small pot of damp soil?
    a. *The faeces were placed onto a pot of dampened soil, in an attempt to replicate how the faeces would be encountered by flies in the wild. Amended (L453)*
15. Lines 264-266 - run on sentence. Actual photos of the traps would be better than the casual drawings.
    a. *Please see point 2, above (p. 2)*
16. Was GC-EAG done on only 4 flies?
    a. *GC-EAG data from only four flies is presented here. GC-EAG is a notoriously difficult technique, and it is usual to present data from only a few repetitions. This is for several reasons: often background noise or insufficient contact of the antennae/electrodes prevents meaningful analysis and repetitions must be discarded, but numerous repeats are not possible because of limited overall volume of (headspace) sample extract (which is finite). Further, nerve cell depolarisation is a binary response (present/absent) with a low false positive rate, therefore only a few repetitions are required to verify that the insect/arthropod in question does indeed respond to that stimulus/chemical. In this study, we were further constrained by fly numbers due to issues of colony longevity (rearing flies on artificial media was not sustainable in the long term), which we have now included (L462-463)*

**Results**

Reviewer #1:

17. Results are weakly stated with relation to the stats.
    a. *Thank you for this constructive comment. We have reviewed the whole results section, re-phrasing where necessary to clarify our meaning.*
18. Lines 302-303 - For feces baits other than horse and sheep, > 50% of flies collected were gravid.
    b. *Amended (L561-562)*
19. Lines 303-305 - run on sentence. Too long.
    c. *Amended (L562-563)*
20. Figure 2 might be more effective in tabular form with means and standard deviation show along with letters indicating statistical separation of means.
    - *While we appreciate this constructive comment, we believe that the data in Figure 2 is best presented in its current, graphical, form, and hope that this will be agreeable to the Editor*
21. Tables need more complete titles.
    d. *Amended*

**Conclusions**

Reviewer #1:

Data basically support conclusions.

22. Limitations of analysis not mentioned.
    a. *We acknowledge and apologise for this omission, and have now included a paragraph on the limitations of the study (L786-807)*
23. Authors discuss how data might be helpful. Authors do not mention the possibility of feces management. This might be difficult to impossible, but people need to know that these are sources of the flies that are transmitting disease to them and their children.
    b. *We thank the reviewer for this thoughtful comment, referring to a hugely important aspect of the biology/control of these flies. However, we want to retain the focus of the paper on the development of an odour-baited trap, exploiting the strong attraction of* M. sorbens *to faeces odour, and investigating the possible mechanism for this attraction. Although relevant, a full discussion of control measures including larval/breeding source management is, unfortunately, beyond the scope of this paper.*
24. Lines 384-387 and lines 405-408 - run on sentences. Too long.
    c. *Amended (L784-786)*

**Editorial and Data Presentation Modifications?**

25. Reviewer #1: The paper could use a thorough revision for clarity and organization. I think the science is basically ok, but the paper can be difficult to read in some places. I have tried to attach a scan of the paper so more of my comments can be seen. I would call this revision minor.
    a. *Thank you very much for this extremely detailed review. We have incorporated your scanned edits throughout, as far as possible.*

**Summary and General Comments**

26. Reviewer #1: The study is a very simple one, but very important for determining the breeding materials used by this fly. Also, the evaluation of chemical compounds found in the feces might be developed into efficacious baits. The sentence structure needs to be tightened up and things more clearly stated.
    a. *We hope that the edits we have made throughout, particularly focussed around the methods/results, have sufficiently clarified the manuscript.*

## Reviewer 2

**Methods**

**Results**

**Conclusions**

**Editorial and Data Presentation Modifications?**

**Summary and General Comments**

Reviewer #2: This is an excellent paper overall. The writing is impeccable. With the exception of a single typographical error on line 202 (~~bais for bias~~) I did not find any errors throughout the manuscript. The work is important, and the degree of replication in different study sites sufficient to make the results quite convincing. A few minor comments for the authors consideration:

27. A photograph of the assembled trap would be helpful, either instead of or supplemental to the schematic drawing.
    a. *In response to this comment and those of the other reviewers, we have updated figure one to include dimensions of the trap as well as a photo as suggested.*
28. Most of the flies collected from Boiram were M*. domestica*. Although the focus of the paper was on *M. sorbens*, it would be interesting to see the collection results for *domestica* as well. How were the house fly collections distributed? Was the pattern substantially different from that of *M. sorbens*?
    a. *We have now included the* M. domestica *trapping data as a supplementary figure S5. Although human faeces were a successful bait, the very strong preference observed in* M. sorbens *is not seen with* M. domestica. *(L618-619)*
29. The climate information is mentioned in the Methods but never mentioned again except for some entries in Table that I was unable to decode. Suggest removing the climate data or discussing it in the context of the collection data.
    a. *We have now expanded the climate information in the results, adding a paragraph that also describes the trap catch of male* M. sorbens *(L695-701).*
30. The number of flies examined by EAG seems rather small. Although I have no expertise with this type of work I wonder whether an EAG response by two flies is sufficient to make conclusions about the attractiveness of the chemical constituents.
    a. *Please see our response to point 16 of reviewer one, who had the same concerns.*

## Reviewer 3

**Methods**

Reviewer #3: Well done authors!

This is an important piece of work which is well over-due. The objectives and methods are clearly stated and described and the interpretation appropriate. This represents a HUGE amount of work!!

I have a couple of minor comments and noticed a few typos

Major comments:

None

Minor comments:

31. In the title and throughout the ms the authors use the term "putative trachoma vector". I suggest removing the word 'putative' as there is no longer any reasonable question as to whether *M. sorbens* is a trachoma vector. *M. sorbens* is a mechanical vector of trachoma, it does not however, have an obligatory role in the transmission of trachoma as Aedes mosquitoes do in the transmission of human Yellow Fever and Dengue. It would be worth mentioning this in the introduction. The relative importance of eye-seeking flies in trachoma transmission will likely vary in time and space. Controlling eye-seeking flies will not block trachoma transmission. Stopping fly-eye contact will not block trachoma transmission.
    a. *Thank you for this well-informed and supportive comment. We are in full agreement that in certain localities, for example The Gambia where this study is partially set, the role of* M. sorbens *in contributing to transmission is unquestionable – as evidenced by gold-standard, randomised controlled trials. However, there is undoubtedly a paucity of studies around the role of* M. sorbens *in the transmission of trachoma, and we are aware that this leads some trachoma authorities to question the role of ‘vectored’ trachoma in other endemic regions. Because of this, we feel that referring to* M. sorbens *as a ‘putative’ vector may be best received, in this instance. However, we continue to conduct extensive studies on the transmission of trachoma by* M. sorbens *and hope to publish those findings in the near future. We believe that these studies considerably expand knowledge concerning the role of* M. sorbens *as a mechanical vector of Ct.*
32. The findings of the work do not identify the role of the electrophysiologically active semio-chemicals identified. It is reasonable to assume that they are attractants for oviposition, since their presence was associated with a convincingly high proportion of gravid female sorbens. However, the presence of an electrophysiological response does not in itself predict behaviour. The compounds may act individually as attractants or repellents, or in combination elicit a different response. This should be the focus of future work
    b. *Again, thank you for this constructive comment, which should have been addressed in the manuscript. We have added a paragraph explaining that EAG responses cannot be interpreted behaviourally in the absence of further work/bioassays (L810-815).*

Typos and other nit-picking:

33. Lines 127-130: Starting 'It is unknown however, ..." the sentence is presented as an 'either/or' construction, when both are reasonable suggestions. I would suggest constructing the sentence to be 'The more prolific emergence could be a result of better larval survival, more eggs being laid as a result of its greater relative attractiveness to ovipositing flies or other factors'. For me I'd continue to consider it as the preferred and 'better' medium for larval development due to the size of the flies emerging after pupation.
    a. *We agree with this comment and have amended our sentence as suggested (L137-140)*
34. Line 145: Repetition of The Gambia
    a. *Amended (L171)*
35. Line 217: Suggest defining gravidity for the lay-audience and the specific context. It is typically used to mean 'mated with developed eggs', was the stage of gravidity estimated?
    a. *Amended. Unfortunately, in this study, the stage of gravidity was not estimated (L406)*
36. Line 300: Any idea what the 'unidentified Musca spp' were in Oromia? That's intriguing. Any idea if these unidentified species are caught from human eyes too?
    a. *At that time, while fly-eye contacts were manually scored, no fly-face captures were made. As far as it was possible to tell without catching, the fly-eye contacts were made by M. sorbens and more unusually M. domestica. The identity of the trapped ‘*Musca’ *species is not known.*
37. Line 306: Do you mean 'per night' or per 24hr period?
    a. *Amended, L603, L1047-1048*
38. Line 379: "...a preference for non-herbivore faeces." made me chuckle. Surely the preference is for "the faeces of non-herbivores"?
    a. *☺ Amended, L778*

**Results**

**Conclusions**

**Editorial and Data Presentation Modifications?**

**Summary and General Comments**
